# Characteristics, health care utilization and cost of patients hospitalized with heart failure

**DOI:** 10.3389/frhs.2025.1571367

**Published:** 2025-04-24

**Authors:** Hanna Winkler, Dorothee Riedlinger, Andrea Figura, Liane Schenk, Martin Möckel, Thomas Reinhold

**Affiliations:** ^1^ Institute of Social Medicine, Epidemiology and Health Economics, Charité—Universitätsmedizin Berlin, Corporate Member of Freie Universität Berlin, Humboldt-Universität zu Berlin and Berlin Institute of Health, Berlin, Germany; ^2^ Division of Emergency Medicine, Campus Virchow-Klinikum and Campus Charité Mitte, Charité—Universitätsmedizin Berlin, Corporate Member of Freie Universität Berlin, Humboldt-Universität zu Berlin and Berlin Institute of Health, Berlin, Germany; ^3^ Department of Psychosomatic Medicine, Charité—Universitätsmedizin Berlin, Corporate Member of Freie Universität Berlin, Humboldt-Universität zu Berlin and Berlin Institute of Health, Berlin, Germany; ^4^ Institute of Medical Sociology and Rehabilitation Science, Charité—Universitätsmedizin Berlin, Corporate Member of Freie Universität Berlin, Humboldt-Universität zu Berlin and Berlin Institute of Health, Berlin, Germany

**Keywords:** heart failure, health care costs, health care utilization, hospitalization, health claims data

## Abstract

**Background:**

Hospitalizations in patients with heart failure are common and their frequency increases with severity of disease. To provide optimal care to these high-risk patients, it is important to know their characteristics and health care utilization patterns.

**Methods:**

This secondary data analysis of the EMANet data set used data from the hospital information system (HIS) of eight hospitals from the center of Berlin to identify patients with heart failure having had at least one hospital treatment during the year 2016. To evaluate the cumulative costs and associated health care utilization in patients with heart failure in 2016 HIS data was linked to individual health claims data from one statutory health insurance fund.

**Results:**

We analyzed health claims data from 970 patients with heart failure (43.4% female; mean age 74.4 years). The mortality rate per year was high at 23.9%. Total annual health care costs from the perspective of the statutory health insurance fund amounted to € 33,668 per patient in 2016. About 69% of total costs arose from hospital treatments. On average, patients spent 37 days in hospital. Ten days of these were caused by unplanned cardiovascular hospitalizations. The utilization of continuous outpatient care by a general practitioner or a cardiologist and a continuous prescription of guideline-based medication is associated with a reduction in the loss of lifetime due to hospitalizations or death.

**Conclusions:**

Patients hospitalized with heart failure have a high burden of morbidity and mortality, which results in a high level of health care costs. A large increase in health care costs and resource use relates to increasing severity of heart failure. Continuous outpatient care may reduce the burden of disease as well as health care costs.

## Background

Heart failure (HF) is one of the most common reasons for inpatient treatment in Germany (1). The most common reason for hospitalizations in patients with chronic heart failure is acute cardiac decompensation: the sudden onset or the deterioration of heart failure symptoms. Hospital mortality of acute cardiac decompensation amounts to 8%–10% (2). From a health economic perspective, heart failure is highly relevant, since the disease-related economic burden is comparable to the total costs of diabetes mellitus or stroke care, although these diseases have a much higher prevalence. In 2020 the costs of heart failure amounted to 1.7% (7,427 Mill. €) of total costs of diseases in Germany (3). Data on costs and health care utilization of heart failure patients in Germany have rarely been published (4). Only a single study (5) calculated annual health care costs based on health claims data from 2002, comparing the annual health care costs of patients with and without heart failure: heart failure in this study was associated with an 2.3 fold increase of costs. The vast majority of costs (72%) was associated with hospital treatment. Since hospitalizations in heart failure are frequent and cost-intensive, it is desirable to understand risk factors as well as protective determinants that influence the likelihood of hospitalization. Hospitalizations due to heart failure might be preventable by continuous ambulatory care provided by general practitioners, cardiologists and/or internal medicine specialists (6) and guideline-directed medication treatment as well as medication adherence (7, 8).

The aim of this study was to evaluate the cumulative costs and associated health care utilization patterns in patients with heart failure in Germany under consideration of patient characteristics such as disease severity by NYHA (New York Heart Association) classes. In addition, the association between days patients spend alive and out of hospital and health care utilization was examined. This analysis contributes to a better understanding of patterns in health care utilization and the identification of risk factors for potentially preventable medical conditions.

## Methods

This analysis was embedded in the EMANet (Emergency and Acute Medicine network for health care research) project primarily aiming to establish a sustainable interdisciplinary health services research structure in emergency and acute medicine with a focus on vulnerable, predominantly older patients suffering from multiple chronic diseases. Mixed-method studies based on primary data collection (surveys, interviews and participant observations) and extraction of secondary data from the hospital information system and from a health insurance fund were conducted in eight emergency departments in the city center of Berlin (Germany) (9). The aim was to investigate specific characteristics and healthcare utilization of acute patients with one of three model diseases groups (hip fracture, respiratory diseases and cardiac symptoms and diseases). Heart failure was one of these target diseases (10). As a sub-study, this analysis aims to present analyses on medical treatment of emergency patients with heart failure.

### Study design and database

This observational study is based on retrospective secondary data (year 2016) from the hospital information system (HIS) of eight hospitals from the center of the German Capital Berlin, supplemented by individual health claims data. The latter were provided by AOK Nordost the participating statutory health insurance fund (SHI). AOK Nordost is the most relevant SHI in the north-eastern part of Germany including federal states Berlin, Brandenburg, and Mecklenburg-Western Pomerania. In 2016, AOK Nordost insured 1.74 million people.

Both data sources were linked on an individual patient level. The linkage was done by a data trustee to ensure data protection requirements. Since there are no standards in data structure and processing for HIS data from emergency departments in Germany, data extraction and harmonization of these HIS data was very complex and time consuming.

All patient data are reflecting the medical routine care in Germany and were not influenced by specific study interventions. Since the analysis was based on anonymized data, patient consent was not required. An approval by the institutional ethics committee has been obtained for the sub-study on patients with cardiac symptoms (EA1/363/16). The HIS data include data of the emergency departments and hospital data in case of inpatient treatment. The routine data documentation in EDs and on wards may be conducted in varying information systems. Data were combined for the whole length of stay by unique identifying codes on patient level. Thus, HIS data contain information on transportation to emergency department, triage category [according to Manchester Triage system (MTS)], symptoms, vital signs, laboratory parameters and diagnoses [coded according to the International Classification of Diseases (ICD) version 10]. The health claims data contains information on demographics, all-cause mortality, inpatient and ambulatory care treatment including all diagnoses, therapeutic appliances, prescribed health care products, rehabilitation treatment, medications, and home health care. For individual consumption of health services, the associated costs from the insurance perspective were available.

### Study population

Patients were identified using the routine HIS data of participating hospitals covering the whole year of 2016. Heart failure was identified by the four-digit diagnosis code “I50.1” of the International Statistical Classification of Diseases and Related Health Problems. 10. Revision. German Modification (ICD-10-GM) (11). This diagnosis was documented either as emergency diagnosis or hospital diagnosis in HIS data. So, the data set included all patients having at least one contact to an emergency department caused by cardiac symptoms and diseases. The study population included all patients (age 18 and older) with an emergency department (ED) visit due to heart failure.

Specific NYHA classes were derived using the fifth digit of the ICD-10-GM code (for example: I50.11 coded NYHA class I). On admission to hospital, this information was coded as part of the emergency diagnoses in the hospital information system (HIS data). As ICD-10-GM diagnosis coding is a relevant basis for remuneration and financing of the German health care system, the information was considered complete and valid. Patients with more than one NYHA class documented in HIS data were assigned to the highest NYHA class documented in 2016. Biodemographic information available from the health claims data included age, sex, and date of death for all causes of mortality. Health claims data were also used to determine whether the patients received nursing care services from the statutory long-term care insurance. This can be considered as an indicator of care dependence, which we assumed to be associated with a considerable impairment in the activities of daily living.

To assess internal validity, the distribution of age, sex and NYHA classes were compared between the total population of all patients with heart failure treated in one of the eight emergency departments in the center of Berlin (Germany) and the subsample of patients for which health claims data were available.

### Parameters of outpatient treatment—medication, diagnoses, utilization patterns

Drug prescriptions and outpatient treatment based on health claims data were evaluated at the quarterly level, as documentation and reimbursement are performed on a quarterly basis in Germany. Thus, the continuity of primary care use was defined as the proportion of quarters in 2016 in which patients visited a general practitioner (GP) at least once in relation to the quarters patient lived (to account for deceased patients). High continuity of care was assumed if patients who did not die within 2016 had at least one GP contact in 3 out of 4 quarters. For patients who died within the year 2016, the number of quarters with GP visits had to equal the number of completed life quarters to assume continuity of care. The continuity of the cardiological outpatient treatment was defined likewise.

Prescribed medications were considered as “HF-related” if they matched a predefined list of substance classes recommended by the guidelines of the European Society of Cardiology that were state-of-the-art in 2016 (12): angiotensin receptor blockers (ARBs), beta blockers, diuretics, Angiotensin-converting-enzyme (ACE) inhibitors, ivabradine. Medication was classified using the Anatomical Therapeutic Chemical Classification (ATC) System codes. The frequency and quantity of actual medication intake could not be observed in the data. However, information on the date of prescription is available. Since medications for chronic diseases in Germany are usually prescribed for a period of 90 days, continuous medication was assumed if patients received a prescription in at least 3 out of the 4 quarters in 2016. For patients who died in 2016, “continuous medication” was assumed if patients received a prescription in all quarters they lived.

Comorbidities were identified in inpatient and outpatient diagnosis data and included the following diagnoses: nicotine dependence, obesity, ischemic heart disease, chronic kidney disease (CKD), chronic obstructive pulmonary disease (COPD), hypertension, atrial fibrillation, diabetes mellitus (DM) and disorders of lipoprotein metabolism. A diagnosis was assumed as confirmed if it was found in at least once in inpatient diagnosis data or in two quarters in 2016 in outpatient diagnosis data according to the recommendation for validation of outpatient diagnosis health claims data in Germany (13).

### Hospitalization and burden of disease

The number of hospitalizations and days spent in hospital during the observation period were evaluated. In addition to the all-cause hospitalizations, the unplanned stays for cardiovascular reasons were analyzed. Unplanned stays were defined as hospitalizations for which the information “emergency” was coded as the reason for admission. Additionally, the discharge diagnosis was used to assess whether a hospital stay was attributable to a cardiovascular condition.

In addition, the days spent alive and out of hospital (DAOH) were calculated based on the date of death and the information on inpatient treatment for the period of one year. This outcome measure is often used in heart failure trials since it combines aspects of mortality and morbidity which are fundamental to the objective of heart failure treatment (14). As an inverse, days lost due to hospitalizations or death were calculated.

### Cost analysis

Total costs consist of the health care costs in 2016. The total health care costs include the costs for hospital treatment, outpatient medical treatment, therapeutic appliances, health care products, rehabilitation treatment, medications, home nursing and transportation costs. The amount of each type of costs was calculated based on the individual patient data provided by the SHI.

In this context, therapeutic appliances comprise outpatient physiotherapy, occupational therapy, speech therapy, nutritional therapy, and podiatry. The term “health care products” was used to describe items that compensate for disabilities and support rehabilitation, such as visual, walking or hearing aids.

The cost category “home nursing” contains only medical care services (e.g., wound care and administration of medication). Basic care such as personal hygiene or food preparation were not included in this category. Transportation costs consist mainly of costs for ambulance transport and emergency service.

### Statistical analysis

Categorical variables were described by their absolute (*n*) and relative frequencies (%). For Continuous normally distributed variables the mean and standard deviation (SD), for skewed distributed continuous variables the median and the interquartile range (IQR) were calculated.

The association (influence of demographic characteristics, comorbidities, and characteristics of health care utilization on days patients lost due to hospitalizations or death was investigated. Since the distribution of this parameter was very skewed, a generalized linear model with gamma distribution and log link function was used. For all statistical analyses, SPSS version 27 (IBM Inc.) was used, and the results of the gamma regression were verified by using R version 4.0.3.

## Results

### Patients' characteristics

**Figure 1:**
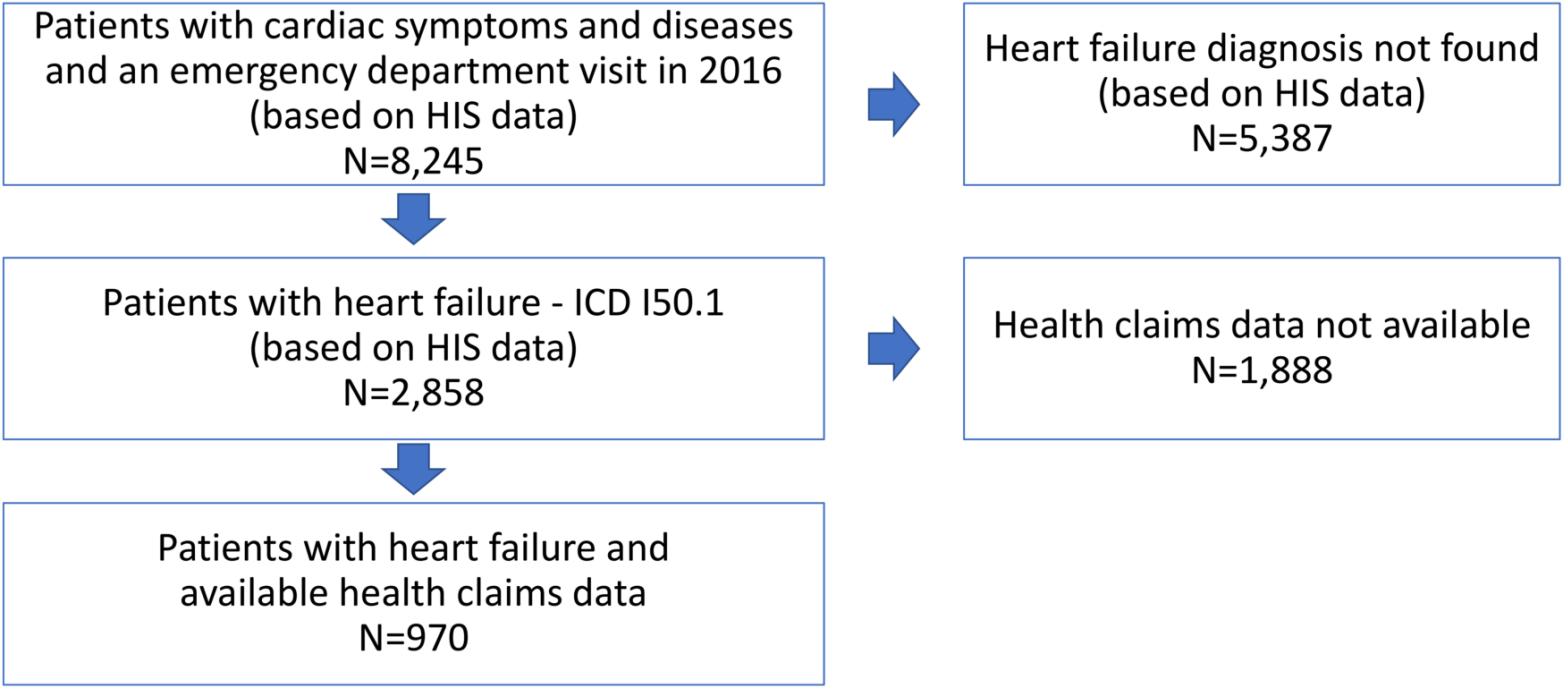
Flow chart.

The initial dataset contained 8,245 patients with cardiac symptoms and diseases who were treated in one of the eight emergency departments in the center of Berlin (Germany) in 2016. Of those, 2,858 patients had a diagnosis of chronic heart failure according to their emergency diagnoses or hospital diagnoses and were eligible for the analysis. For 970 of these patients, health claims data from a statutory health insurance fund were available in addition to HIS data (see Figure 1). The average mean age of ED patients with HF was 72.5 years and 42.1% of them were female.: NYHA I was diagnosed in 9.6% of the patients, NYHA II in 23.7% with, NYHA III in 33.5%, and NYHA IV in 30.4%. No NYHA class was specified for 2.8% of the population.

The following results are based on the SHI population of 970 patients (female 43.4%; 74.4 ± 12.5 years) with chronic heart failure (Table 1). The most common comorbidities of this population were hypertension (90.1%), ischemic heart disease (73.4%), disorders of lipoprotein metabolism (69.2%) and chronic kidney disease (62.8%). The all-cause mortality rate in 2016 was 23.9%. Another 12.7% of the population died in 2017. It was found that 51.1% of the patients were care dependent and received nursing care services in 2016.

**Table 1:** Patient characteristics.

Characteristics	Total = 970	
Demographic data	Mean	SD
Age, years	74.4	12.5
Days alive and out of hospital (DAOH)	294.9	83.8
	Number	Percentage
Death in 2016	232	23.9%
Death in 2017	123	12.7%
Care dependence	496	51.1%
Sex		
Male	549	56.6%
Female	421	43.4%
Heart failure classification		
NYHA I	102	10.5%
NYHA II	203	20.9%
NYHA III	333	34.3%
NYHA IV	302	31.1%
Unkown/Not specified	30	3.1%
Comorbidities^a^		
Hypertension (I10)	874	90.1%
Ischemic heart disease (I20–I25)	712	73.4%
Disorders of lipoprotein metabolism (E78)	671	69.2%
Chronic kidney disease (N18)	609	62.8%
Atrial fibrillation (I48)	524	54.0%
Diabetes mellitus (E11)	505	52.1%
Chronic obstructive pulmonary disease (J44)	382	39.4%
Obesity (E66)	325	33.5%
Nicotine dependence (F17)	162	16.7%
Continuous medication		
Beta blockers	525	54.1%
Diuretics^b^	473	48.8%
Angiotensin-converting-enzyme (ACE) inhibitors	307	31.6%
Angiotensin receptor blockers (ARBs)	107	11.0%
Ivabradine	6	0.6%
No HI-specific medication	49	5.1%

^a^ Multiple options possible.

^b^ Diuretics contained aldosterone antagonists, loop diuretics and thiazides.

One-third of all patients had a NYHA functional class III (34.3%) and another third were in NYHA IV (31.1%). Patients with a higher NYHA class were slightly older and the proportion of females decreased with increasing heart failure severity (see Table 2).

**Table 2:** Patient characteristics differentiated by NYHA classes.

Characteristics	NYHA I (*n* = 102)		NYHA II (*n* = 203)		NYHA III (*n* = 333)		NYHA IV (*n* = 302)		Total (*n* = 970)	
	Mean	SD	Mean	SD	Mean	SD	Mean	SD	Mean	SD
Demographic data										
Age, years	73.6	11.8	72.4	13.0	75.8	12.0	74.4	13.2	74.4	12.5
Days alive and out of hospital	319.6	63.9	312.2	73.0	287.3	87.6	282.2	88.8	294.9	83.8
	*n*	%	*n*	%	*n*	%	*n*	%	*n*	%
Death in 2016	16	15.7%	33	16.3%	88	26.4%	90	29.8%	232	23.9%
Death in 2017	12	11.8%	21	10.3%	50	15.0%	38	12.6%	123	12.7%
Sex										
Male	51	50.0%	118	58.1%	183	55.0%	178	58.9%	549	56.6%
Female	51	50.0%	85	41.9%	150	45.0%	124	41.1%	421	43.4%
Care dependence	46	45.1%	92	45.3%	194	58.3%	150	49.7%	496	51.1%
Outpatient Treatment										
Continuous care by a GP	81	79.4%	183	90.1%	302	90.7%	258	85.4%	852	87.8%
Continuous outpatient care by a cardiologist	6	5.9%	11	5.4%	22	6.6%	29	9.6%	73	7.5%
In-patient Treatment	Median	IQR	Median	IQR	Median	IQR	Median	IQR	Median	IQR
Number of hospital stays	2	(1–4)	3	(1–4)	3	(2–5)	3	(2–4)	3	(2–4)
Number of unplanned cardiovascular hospitalizations	0	(0–1)	1	(0–1)	1	(0–1)	1	(0–1)	1	(0–1)
	Mean	SD	Mean	SD	Mean	SD	Mean	SD	Mean	SD
Number of days spent in hospital	26.9	29.7	32.5	36.5	40.6	41.4	39.8	35.0	37.2	38.1
Number of days of unplanned cardiovascular hospitalizations	4.1	6.9	6.7	13.8	9.9	14.0	13.5	17.1	9.6	14.7

### Resource consumption and costs

While the vast majority of patients (87.7%) have received continuous care by a General Practitioner (GP), only a few patients (7.5%) have utilized continuous outpatient care from a cardiologist. However, the number of patients with continuous contact to a cardiologist increased with the severity of heart failure.

Medication prescriptions for at least one guideline-recommended drug agent were given to almost all patients. Beta blockers were prescribed to 54.1% of the patients, 48.8% received diuretics, 31.6% got ACE inhibitors and 11.0% were treated by AT1 receptor antagonists, but 5.1% of the patients did not receive any heart-failure specific medication prescription.

Patients spend on average 37.2 days in a hospital in 2016. The median number of unplanned hospital stays was 3 (2–4) and for unplanned cardiovascular hospitalizations 1 (0–1), respectively. Patients with higher NYHA class had a higher number and duration in both total and unplanned cardiovascular hospital stays. One third of the hospital days of patients with NYHA class IV were caused by unplanned cardiovascular hospitalizations, for all NYHA classes the share of unplanned cardiovascular hospitalizations amounted to 26%.

In 2016 patients spent on average 295 days alive and out of hospital (DAOH). This implies that they have lost more than 2 months (71 days) due to premature death or hospitalization (all causes).

**Figure 2:**
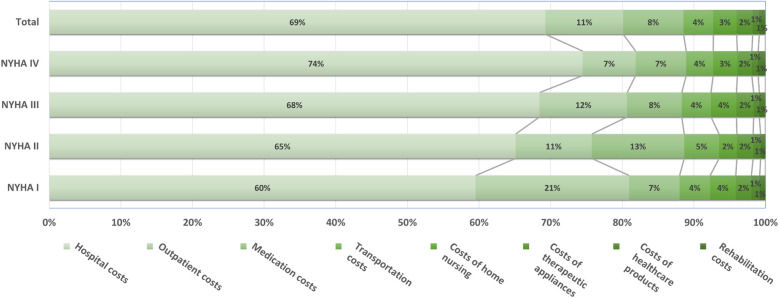
Relative health care costs differentiated by NYHA classes.

**Figure 3:**
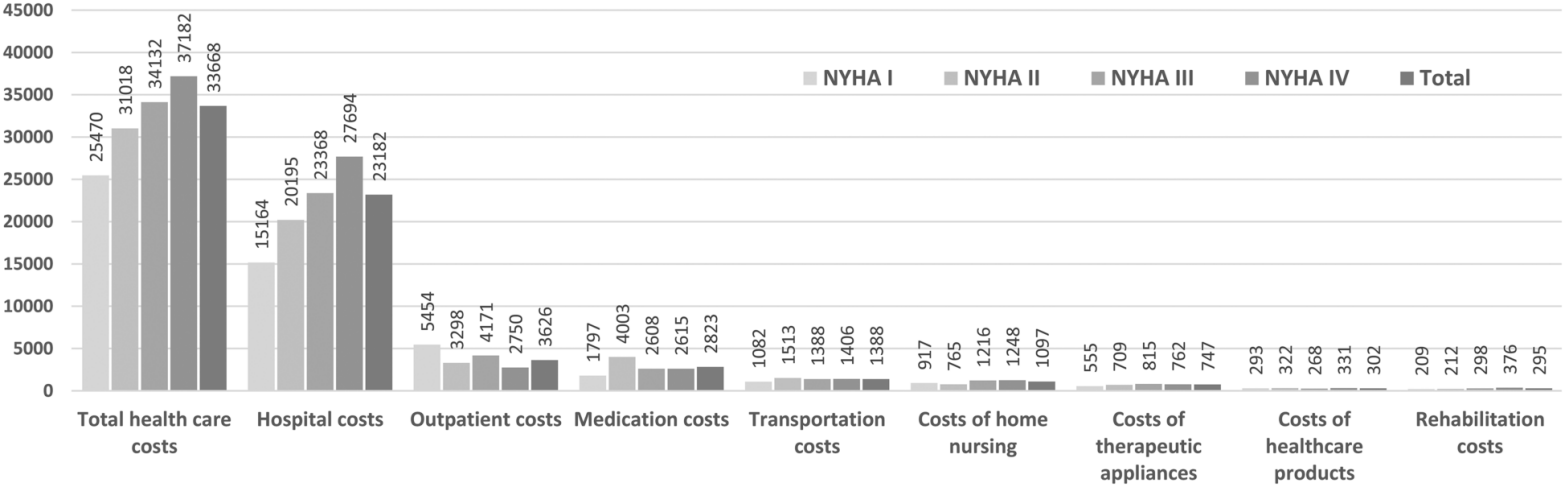
Absolute annual health care costs in € differentiated by NYHA classes.

The average annual patient related total health care costs of the study population amounted to € 33,668. The higher the NYHA class the more the costs increased (see Figure 2) and was 1.5 times higher in patients diagnosed with NYHA class IV compared to patients with NYHA class I. The largest cost category in all NYHA classes were hospital costs with € 23,347, representing 69% of the total costs (see Figure 3). Outpatient costs were found to be the second largest cost category with on average € 3,626. While the share of hospital costs increases with disease severity and amounts to 74% in NYHA class IV, the share of outpatient costs on the total costs are highest in patients with NYHA class I. Medication costs represented the third largest cost category at € 2,823 and patients with NYHA class II had the highest average medication costs. Transportation costs are the fourth highest cost category, accounting for 4% of the total costs. Costs of home nursing care, rehabilitation costs, costs of therapeutic appliances and costs of healthcare products accounted for a comparatively small proportion of the total costs. No relevant differences between the NYHA classes were observed for these cost categories.

### Regression analysis

Regression analysis revealed different factors that are potentially associated with a reduction of days patients lost due to hospitalizations or death. A negative correlation existed between continuous prescription of angiotensin receptor blockers (ARBs), beta blockers, diuretics or ACE inhibitors and the loss of DAOH. Continuous care by a GP is related to a statistically significant decrease in the days patients lost (*p* = 0.005). The same applies to continuous outpatient care by a cardiologist (*p* = 0.020). Compared to patients without continuous care those patients (with continuous care) lost approximately 7 days less of lifetime over the observation period.

The following factors were as to an increase in days patients lost due to hospitalizations or death and thus shorten lifespan: higher age, care dependence, the severity of heart failure measured by NYHA classes. Transforming the regression coefficient to an estimated mean reveals that an increase of one NYHA class is associated with a loss of about 13 days of life. Additionally, the comorbidities diabetes mellitus, chronic kidney disease and atrial fibrillation are related to a loss of DAOH.

Furthermore, the analysis showed that there is no influence of sex on the outcome parameters days lost due to hospitalization or death. Table 3 displays the results of the regression analysis.

**Table 3:** Impact of morbidity, medication and outpatient health care utilization characteristics on days lost due to hospitalization or death with confidence intervals and *p* values using gamma regression.

Characteristics	Regression coefficent	Std. error	Asymptotic lower 95% CI	Asymptotic upper 95% CI	*P*-value
Intercept	2.327	0.274	1.790	2.864	<0.001
Sex (male)	0.123	0.069	−0.012	0.257	0.074
Age	0.015	0.003	0.009	0.022	<0.001
Care dependence	0.572	0.074	0.427	0.717	<0.001
NYHA class	0.222	0.034	0.155	0.289	<0.001
Obesity	0.085	0.074	−0.059	0.230	0.246
Nicotine dependence	0.143	0.094	−0.042	0.327	0.130
Diabetes mellitus	0.187	0.070	0.049	0.325	0.008
Chronic kidney disease	0.192	0.072	0.052	0.332	0.007
Chronic obstructive pulmonary disease	0.052	0.067	−0.080	0.183	0.442
Hypertension	0.017	0.118	−0.215	0.249	0.888
Ischemic heart disease	0.011	0.078	−0.141	0.164	0.886
Disorders of lipoprotein metabolism	−0.236	0.077	−0.387	−0.084	0.002
Atrial fibrillation	0.242	0.067	0.111	0.373	<0.001
Continuous prescription of angiotensin receptor blockers	−0.404	0.109	−0.618	−0.190	<0.001
Continuous prescription of beta blockers	−0.317	0.074	−0.462	−0.173	<0.001
Continuous prescription of diuretics	−0.154	0.072	−0.296	−0.013	0.033
Continuous prescription of ACE inhibitors	−0.333	0.078	−0.485	−0.181	<0.001
No HF-related medication	0.196	0.159	−0.116	0.509	0.217
Continuous care by a GP	−0.300	0.106	−0.507	−0.092	0.005
Continuous outpatient care by a cardiologist	−0.295	0.127	−0.544	−0.045	0.020

## Discussion

In our study cohort of patients hospitalized with heart failure in 2016 health care utilization and costs increased with respect to the severity of disease. Hospital costs clearly accounted for the largest share of total costs, followed by medication cost and outpatient costs. The analysis of the burden of disease showed that patients have lost more than two months of lifespan due to hospitalizations or death. This incorporates both a high mortality rate and a large number of days in hospital in this population. While advanced age and higher NYHA class, as well as the presence of diabetes mellitus, chronic kidney disease and atrial fibrillation are associated with higher loss of DAOH, continuous drug therapy with angiotensin receptor blockers, beta blockers, diuretics or ACE inhibitors appeared to reduce the loss of DAOH. The present analysis indicates that a continuous care by a cardiologist but also by a GP decreases the loss of DAOH.

From epidemiological studies (15) it is known that the proportion of females with heart failure in Germany is larger (55.0%) and the mean age is higher (76.2 years) in comparison to the presented results. The lower proportion of women in the study population compared to the general population in Germany may be attributed to the fact that women have lower hospitalization rates than men (1). The comparable younger population in this analysis may reflect the lower age of residents of urban areas (16).

The results on the distribution of costs are consistent with previous analyses from Germany (5, 17, 18) and Spain (19). All sources find a share of inpatient costs on total costs of about 70%. Zugck et al. (5) reported the share of hospital costs as 72%, the share of medication costs as 11% and the share of outpatient costs as 4% for 2002 which is still comparable to the results of our present study. Interestingly, no other recent cost analyses from Germany and no cost differentiation by NYHA class was available on PubMed search.

Of note, medication costs were highest in NYHA class II. The reason for this is a small number of high-cost cases with annual medication costs above 40,000 € in this group. These high-cost cases were unrelated to heart failure drug therapy, but were caused for instance, by treatment with cytostatic drugs. The comparatively higher proportion of outpatient costs in NYHA class I may be explained by the higher number of days patients spent alive and out of the hospital compared to patients in higher NYHA classes.

The comparison of the distribution of age, sex and NYHA classes between the total population and the subsample of patients for which health claims data were available, showed no relevant differences Thus, it can be assumed that the results apply for the total population, too.

A strength of the presented study lies in the linkage of data from emergency departments and health claims data—which is unique for German health care analyses where data sources normally are strictly divided between the health care sectors. Hence, a more reliable selection of patients based on emergency diagnosis data compared to a selection based solely on health claims data was possible. The study provided differentiated insight into the cost distribution of hospitalized heart failure patients and showed the association between health care utilization characteristics and the endpoint days lost due to hospitalization or death, which is important in current heart failure research.

## Limitations

Limitations of this investigation include those associated with secondary data analyses and especially health claims data studies for example in terms of the reliance on accurate coding and the lack of clinical data for the course of the hospital stay or out-patient care. Due to the specific setting of data acquisition in emergency departments in the center of Berlin, the validity of the results is limited to hospitalized patients in metropolitan areas in Germany. Furthermore, the calculated costs are only valid from the perspective of statutory health insurance in Germany since these costs depend on the administered prices of the underlying reimbursement systems and are not comparable to market prices. Variations in days patients lost due to hospitalizations or death can not only be related to the characteristics that were used.

Moreover, the study design of an observational study allows to identify associations but not to prove causality.

Since there are still no uniform standards for HIS data management in Germany, the data acquisition process as well as the data delivery to the eight different emergency departments and the harmonization of all delivered data sets was very complex. This resulted in a considerable time lag between data generation and data analysis. Since data was collected in 2016, it must be noted that drug therapy has changed significantly in the meantime due to the introduction of angiotensin receptor neprilysin inhibitors (ARNI) and Sodium-glucose co-transporter-2 (SGLT-2) inhibitors followed by the adaptation of the European heart failure guidelines. Thus, the results regarding drug therapy may not reflect the current state and could consequently have changed costs and utilization patterns. Only the study of Michel et al. (20) is known for Germany, which used slightly more recent data (2016–2019) on drug therapy patterns. The two new substances still played a minor role in this period. The results on mortality and hospitalizations were comparable to those presented here. Unfortunately, no results are currently known for the (post-)pandemic period (after 2019), so it remains unclear whether and to what extent the characteristics, utilization patterns, and costs of heart failure have changed.

For this reason, there is a high need for similarly targeted studies with more current data. Future research should investigate whether and how the extension of therapeutic options in recent decades and the pandemic-related changes have affected utilization patterns and costs of heart failure patients. In addition, a longer data period (more than one year) would be desirable for future studies, as changes in utilization can be better depicted with regard to the progression of heart failure.

The present study can be seen as preliminary work and provides a foundation for assessing future results.

## Conclusion

The presented study provides an approximation of the annual health care costs for patients with heart failure according to the severity of the disease. Higher severity of heart failure was associated with higher health care costs. This applied in particular to the hospital costs. Mortality was high and patients spent many days in hospital. Results suggest that continuous outpatient care by a GP as well as by a cardiologist and continuous drug therapy are associated with less days patients lost due to hospitalizations or death. To date, analyses on this topic in Germany are scarce and further investigations are necessary to validate the results.

## Data Availability

The datasets presented in this article are not readily available because based on the data protection concept, positively evaluated by the Charité data protection office in 2017, the data set of HIS data cannot be published. To fulfil the legal requirements to obtain health claims data in Germany, researchers must receive permission for a specific research question from the German Federal Office for Social Security. Requests to access the datasets should be directed to hanna.winkler@charite.de.
